# Analysis of the current status and influencing factors of cross-regional hospitalization services utilization by basic medical insurance participants in China − taking a central province as an example

**DOI:** 10.3389/fpubh.2023.1246982

**Published:** 2023-12-18

**Authors:** Tianyu Song, Rongfei Ma, Xiange Zhang, Bangliang Lv, Zihang Li, Mengzi Guo, Min Yuan, Zhiru Tang

**Affiliations:** School of Health Service and Management, Anhui Medical University, Hefei, Anhui, China

**Keywords:** cross-regional hospitalization, basic medical insurance, hospitalization expense, actual reimbursement rate, exploratory factor analysis

## Abstract

**Background:**

The geographically uneven distribution of healthcare resources has resulted in a dramatic increase of cross-regional hospitalization services in China. The over-use of cross-regional hospitalization services may hinder the utilization and improvement of local hospitalization services. It is of great practical significance to study the utilization of cross-regional hospitalization services and its influencing factors in order to effectively allocate medical resources and guide patients to seek medical treatment rationally. Therefore, this study aims to analyze the current situation and influencing factors of the utilization of cross-regional hospitalization services by patients insured by basic medical insurance in China.

**Methods:**

A total of 3,291 cross-provincial inpatients were randomly selected in a central province of China in 2020. The level of medical institutions, hospitalization expenses and actual reimbursement rate were selected as indicators of hospitalization service utilization. Exploratory factor analysis was used to assess the dimensionality of influencing factors and reduce the number of variables, and binomial logistic regression analysis and multiple linear regression analysis to explore the influencing factors of the utilization of cross-regional hospitalization services.

**Results:**

The proportion of cross-provincial inpatients choosing tertiary hospitals was the highest with average hospitalization expenses of 24,662 yuan and an actual reimbursement rate of 51.0% on average. Patients insured by Urban Employees’ Basic Medical Insurance (UEBMI) were more frequently (92.9% vs. 88.5%) to choose tertiary hospitals than those insured by Urban and Rural Residents’ Basic Medical Insurance (URRBMI), and their average hospitalization expenses (30,727 yuan) and actual reimbursement rate (68.2%) were relatively higher (*p* < 0.001). The factor “income and security,” “convenience of medical treatment” and “disease severity” had significant effects on inpatients’ selection of medical institution level, hospitalization expenses and actual reimbursement rate, while the factor “demographic characteristics” only had significant effects on hospitalization expenses and actual reimbursement rate.

**Conclusion:**

Cross-provincial inpatients choose tertiary hospitals more frequently, and their financial burdens of medical treatment are heavy. A variety of factors jointly affect the utilization of cross-provincial hospitalization services for insured patients. It is necessary to narrow down the gap of medical treatment between UEBMI and URRBMI patients, and make full use of high-quality medical resources across regions.

## 1 Background

Cross-regional treatment is a widespread medical access phenomenon that reflects the current state of reform and development of health care system in a country or region (1). Many health care systems offer patients the opportunity to be able to move across regions, countries and even continents for medical care. For example, the European Court of Justice (ECJ) developed policies on patient mobility to guarantee the right of European residents to receive health care across European Union (EU) countries (2).The geographically uneven distribution of healthcare resources in many countries and regions has resulted in a noticeable increase of the number of patients treated across regions (3–5).

In China, cross-regional treatment of patients usually refers to the medical behavior of the population insured by basic medical insurance and whose medical treatment location does not coincide with where they are insured (6, 7), including three levels: cross-county, cross-city and cross-province (8).Many countries such as Germany, the United Kingdom and Japan, have established government/compulsory health insurance coverage covering nearly the whole population (9). Similarly, China has also established a basic medical insurance scheme covering more than 95% of the population (10), including two main categories: Urban Employees’ Basic Medical Insurance (UEBMI) and Urban and Rural Residents’ Basic Medical Insurance (URRBMI). UEBMI is designed to compensate urban employees for financial losses due to the risk of illness. URRBMI is used to compensate medical expenses arising from medical care needs of people not covered by UEBMI, including the older adult, children, students, urban, and rural non-employed residents.

Currently, China’s basic medical insurance scheme mainly implements the municipal-level coordination system, which has typical regional characteristics (11), and the phenomenon of cross-regional medical treatment is becoming more and more prevalent in China because of the rapid increase of people’ income, frequent movement of people across regions, as well as the imbalance in the allocation of medical resources among regions. The increasing number of patients treated across regions may hinder the utilization and improvement of local hospitalization services in insurance participating regions, and exacerbate the uneven distribution of medical resources. Therefore, it is of great practical significance to study the utilization of cross-regional hospitalization services and its influencing factors by basic medical insurance participants, in order to rationally allocate medical resources and guide patients to rationally seek medical treatment.

Only several studies were identified researching the utilization of cross-regional hospitalization services. On the one hand, they focused on analyzing the utilization of cross-regional hospitalization services by patients diagnosed with a specific disease such as tumor (6, 8, 12), rare diseases (13), and non-emergency hip arthroplasty (14); on the other hand, they were only restricted to one certain aspect of cross-regional hospitalization service utilization, such as the choice of medical institution level (14, 15), hospitalization expenses and deductibles (16). However, rare research was found analyzing the utilization of cross-regional hospitalization services for patients insured by basic medical insurance from a more comprehensive perspective. Therefore, to fill the gap in the literature, this study takes cross- regional inpatients with UEBMI or URRBMI as the research subjects, analyzes the current situation and influencing factors of the utilization of cross- regional hospitalization services, thereby providing data support for optimizing medical resource allocation and guiding patients to seek medical care rationally.

## 2 Method

### 2.1 Data sources

The example province selected for this study is a typical area with a large number of cross-provincial patients going out for medical treatment and incurring high medical expenses. It is located in central China, and its total expenses on cross-regional medical care in 2020 were 20.97 billion yuan ranking among the top three provinces in China, 10.61 billion yuan of which was for out-of-province medical care (17). The sample data of this study was obtained from the province’s basic medical insurance information system for patients hospitalized for cross-provincial treatment in 2020. Basic medical insurance information system data can be widely used to capture the expenses of inpatient care and provide evidence for targeting priority diseases with heavy economic burdens (18). In this study, multi-stage stratified whole-group random sampling was used. In the first stage, five representative cities were selected based on the scale of population, health resource possession, and geographic location. Following that, one urban district and one county were randomly selected from each city as the sampling areas, and finally, a total of 5,597 patients treated across provinces were sampled at a rate of 5.1%. Patients aged 17 years or older and with principal or other diagnoses meeting the criteria of the International Classification of Diseases −10 codes (ICD-10) were included in the study, and patients missing key data items, such as total hospitalization expenses and reimbursement rates, were excluded.

### 2.2 Statistical analysis

Patients’ characteristics were extracted and viewed as variables as shown in Table 1. Based on relevant references (19), the level of medical institution, hospitalization expenses and actual reimbursement rate were commonly used as indicators of inpatient service utilization. Excluding infant and toddler participants of URRBMI, those aged 17 years and older were selected for the study according to China’s definition of labor force age. Disposable income *per capita*, distance to medical institution and hospitalization days were classified into three levels: low, medium and high. Disease types were categorized into tumor and non-tumor diseases according to ICD-10.

**Table 1:** Variables for statistical analysis.

Variable	Definition	Value assignment
Gender	Patient’s gender	Female = 0, Male = 1
Age	Patient’s age	17 to 44 years old = 0, 45 to 59 years old = 1, 60 years old and above = 2
*Per capita* disposable income	Patient’s *per capita* disposable income	Low(≤ 19,726 yuan) = 0, Medium(19,726 yuan - 34,030 yuan) = 1, High (>34,030 yuan) = 2
Type of medical insurance	Patient’s type of medical insurance	URRBMI = 0,UEBMI = 1
Distance to medical institution	Patient’s distance to medical treatment	Low(≤205.9 km) = 0, Medium(205.9 km-493 km) =1, High(>493 km) =2
Type of disease	Patient’s disease type	Tumor diseases =1, Non-tumor diseases =2
Hospitalization days	Number of days patients stay in hospital	High (>9 days) =0, Medium (5 days-9 days) =1, Low(≤4 days) = 2
Settlement method^*^	Settlement method of patient’s hospitalization expenses when being discharged	Direct settlement = 0. Manual reimbursement = 1
Medical institution level	The hierarchy of medical institutions	Primary and secondary hospitals = 0, Tertiary hospitals = 1
Hospitalization expenses	Patient’s hospitalization expenses	Continuous variables
Actual reimbursement rate	The proportion of the actual reimbursed part to the total hospitalization expenses for an individual patient	Continuous variables

* When patients receive cross-provincial hospitalization services, there are two main ways to settle hospital expenses: immediate reimbursement once discharged (direct settlement); and manual reimbursement at the place where they were insured (manual reimbursement); UEBMI, Urban Employees’ Basic Medical Insurance.

URRBMI: Urban and Rural Residents’ Basic Medical Insurance.

Chi-square tests and one-way ANOVA were used to explore differences in the main demographic characteristics as well as hospitalization service utilization between different patient groups. Exploratory factor analysis was used to assess the dimensionality of factors influencing hospitalization service utilization and to reduce the number of variables. Binomial logistic regression analysis and multiple linear regression analysis were used to explore the factors influencing the utilization of cross-provincial hospitalization services in terms of medical institution level, hospitalization expenses, and actual reimbursement rate. To make the data approximate normal distribution, the hospitalization expenses were log-transformed. SPSS 26.0 was employed as the data processing software and α = 0.05 as the test level.

## 3 Results

### 3.1 Basic characteristics of sampled cross-provincial inpatients

A total of 3,291 samples were included in this study. The results in Table 2 show that the study sample presented a higher percentage in terms of the following characteristics: female, URRBMI, 45–59 years old, low-income, non-tumor diseases, high distance to medical institution, medium hospitalization days, and direct settlement. Compared with patients with URRBMI, those with UEBMI had a higher percentage in terms of male, high disposable income *per capita*, tumor diseases, medium distance to medical institution, low hospital days, and direct settlement, and all of these differences were of statistical significance (*p* < 0.001). The high *per capita* disposable income group occupied the largest percentage of UEBMI patients, while the largest percentage of URRBMI patients was accounted for by the low *per capita* disposable income group.

**Table 2:** Basic characteristics of sampled inpatients.

Variables	Total	UEBMI	URRBMI	*χ*^2^/*Z*	*p*
	(N = 3,291)	(N = 1,008)	(N = 2,283)		
Gender (*n*, %)				12.813	<0.001
Male	1,563(47.5%)	526(52.2%)	1,037(45.4%)		
Female	1728(52.5%)	482(47.8%)	1,246(54.6%)		
Age (*n*, %)				5.014	0.082
17 ~ 44	707(21.5%)	232(23.0%)	474(20.8%)		
45 ~ 59	1,422(43.2%)	407(40.4%)	1,015(44.5%)		
60~	1,162(35.3%)	369(36.6%)	749(34.8%)		
*Per capita* disposable income (*n*, %)				838.192	<0.001
Low	1730(52.6%)	153(15.2%)	1,577(69.1%)		
Medium	474(14.4%)	219(21.7%)	255(11.2%)		
High	1,087(33.0%)	636(63.1%)	451(19.8%)		
Type of disease (*n*, %)				133.995	<0.001
Tumor diseases	1,270(38.6%)	538(53.4%)	732(32.1%)		
Non-tumor diseases	2021(61.4%)	470(46.6%)	1,551(67.9%)		
Distance to medical institution (*n*, %)				347.747	<0.001
Low	1,106(33.6%)	183(18.2%)	923(40.4%)		
Medium	981(29.8%)	519(51.5%)	462(20.2%)		
High	1,204(36.6%)	306(30.4%)	898(39.3%)		
Hospitalization days (*n*, %)				48.184	<0.001
Low	1,112(33.8%)	422(41.9%)	687(30.1%)		
Medium	1,225(37.2%)	306(30.4%)	919(40.3%)		
High	954(29.0%)	278(27.6%)	676(29.6%)		
Settlement method (*n*, %)				76.355	<0.001
Direct settlement	2,273(69.1%)	803(79.7%)	1,470(64.4%)		
Manual reimbursement	1,018(30.9%)	205(20.3%)	813(35.6%)		

### 3.2 Utilization of hospitalization services by cross-provincial patients

Table 3 shows that cross-provincial patients who chose tertiary hospitals accounted for the largest percentage (89.9%), The case-average hospitalization expenses of all patients amounted to 24,662 yuan and the average actual reimbursement rate to 51.0%. There were significant differences between UEBMI and URRBMI cross-provincial patients in the utilization of hospitalization services: the choice of medical institution level (Tertiary hospitals: UEBMI 92.9% > URRBMI 88.5%), case-average hospitalization expenses (UEBMI 30,727 yuan > URRBMI 21,984 yuan), and actual reimbursement rate (UEBMI 68.2% > URRBMI 53.3%).

**Table 3:** Utilization of cross-provincial hospitalization services.

Variables	Total	UEBMI	URRBMI	*χ*^2^/*Z*	*p*
	(*N* = 3,291)	(*N* = 1,008)	(*N* = 2,283)		
Medical institution level (*n*,%)				14.399	<0.001
Primary and secondary hospitals	334(10.1%)	72(7.1%)	262(11.5%)		
Tertiary hospitals	2,957(89.9%)	936(92.9)	2,021(88.5%)		
Hospitalization expenses Mean (SD)	24,662 (36,249.10)	30,727 (43,257.83)	21,984 (32,330.39)	−7.342	<0.001
Actual reimbursement rate Mean (SD)	51.0% (23.0%)	68.2% (18.1%)	53.3% (21.6%)	−26.013	<0.001

### 3.3 Influencing factors of the utilization of hospitalization services By cross-provincial patients

#### 3.3.1 Factor analysis of the influential factors

Given the data features of the information system of basic medical insurance, this study collected a total of eight variables related to the basic characteristics of cross-provincial inpatients and their access to medical treatment, as has been presented in Table 2. The correlation between indicators can cause some bias in the value assignment when data are analyzed. To reduce such effects, factor analysis was therefore adopted. Bartlett’s spherical test showed that chi-square = 1844.716, *p* < 0.001, and KMO test was 0.555, indicating that factor analysis has good adaptability and is suitable to be used in this study.

After the initial common factors were extracted by the principal component method, the initial common factors were rotated by the maximum variance method, and the eigenvalues of the first four common factors were all greater than 1. The cumulative contribution of variance was 65.07%, reflecting most of the information of the eight variables. According to the rotated component matrix as shown in Table 4, the two factors with data greater than 0.45 (type of health insurance and *per capita* disposable income) were named “income and security.” Distance to medical institution and settlement method were named “convenience of medical treatment,” The study named the type of diseases and hospitalization days as “disease severity,” and gender and age as “demographic characteristics.”

**Table 4:** Rotational component matrix of factors influencing cross-provincial hospitalization service utilization.

Variable Content	Factor 1 income and security	Factor 2 convenience of medical treatment	Factor 3 disease severity	Factor 4 demographic characteristics
Gender X_1_	−0.104	−0.148	0.145	0.867
Age X_2_	0.244	0.281	−0.423	0.545
Type of medical insurance X_3_	0.840	−0.029	0.123	−0.013
*Per capita* disposable income X_4_	0.824	−0.028	0.090	0.008
Type of disease X_5_	0.236	−0.063	0.701	0.181
Distance to medical institution X_6_	0.132	0.798	0.026	0.009
Settlement method X_7_	−0.242	0.756	0.103	−0.065
Hospitalization days X_8_	0.044	0.223	0.658	−0.097

The coefficients indicate the positive and negative correlations and weights between each factor and all variables. As shown in Table 5, the score models for the four common factors were derived as:

**Table 5:** Component score coefficient matrix.

Variable	Factor1	Factor2	Factor3	Factor4
Gender	0.19	0.246	−0.395	0.474
Age	−0.144	−0.091	0.193	0.809
Type of medical insurance	0.533	−0.005	−0.003	−0.058
*Per capita* disposable Income	0.525	−0.003	−0.029	−0.039
Type of disease	0.047	−0.062	0.606	0.186
Distance to medical institution	0.103	0.59	−0.024	0.025
Settlement method	−0.149	0.545	0.093	−0.017
Hospitalization days	−0.048	0.137	0.569	−0.053

Factor1 = 0.19X_1_-0.144X_2_ + 0.533X_3_ + 0.525X_4_ + 0.047X_5_ + 0.103X_6_-0.149X_7_-0.048X_8_.

Factor2 = 0.246X_1_-0.091X_2_-0.005X_3_-0.003X_4_-0.062X_5_ + 0.59X_6_ + 0.545X_7_ + 0.137X_8_.

Factor3 = −0.395X_1_ + 0.193X_2_-0.003X_3_-0.029X_4_ + 0.606X_5_-0.024X_6_ + 0.093X_7_ + 0.569X_8_.

Factor4 = 0.474X_1_ + 0.809X_2_-0.058X_3_-0.039X_4_ + 0.186X_5_-0.025X_6_-0.017X_7_-0.053×_8_.

#### 3.3.2 Analysis of influencing factors of medical institution level

Factor 1 (income and security), Factor 2 (convenience of medical treatment), Factor 3 (disease severity), and Factor 4 (demographic characteristics) were set as independent variables, and the level of medical institution as the dependent variable. Logistic regression was used to explore the factors influencing the choice of medical institution level. According to the OR and *p*-values in Table 6, the logistic regression analysis showed that income and security (OR = 1.383 > 1), convenience of medical treatment (OR = 0.503 < 1), and disease severity (OR = 1.314 > 1) significantly influenced the choice of medical institution level, and demographic characteristics had no significant effect on the choice.

**Table 6:** Results of logistic regression analysis on medical institution level.

Variables	β	S.E.	*p*-value	OR	95%C.I. of OR	
					Lower limit	Upper limit
Income and security(F1)	0.325	0.068	0.000	1.383	1.211	1.581
Convenience of medical treatment (F2)	−0.688	0.061	0.000	0.503	0.446	0.567
Disease severity (F3)	0.273	0.063	0.000	1.314	1.161	1.487
Demographic characteristics (F4)	0.079	0.059	0.181	1.083	0.964	1.216

Dependent variable = Medical institution level.

#### 3.3.3 Analysis of influencing factors of hospitalization expenses

To explore the factors influencing hospitalization expenses, multiple linear regression was adopted with Factor 1 to 4 as independent variables and the logarithm of hospitalization expenses as the dependent variable. The results of multiple linear regression are given in Table 7. The results show that income and security (β = 0.183 > 0), convenience of medical treatment (β = −0.102 < 0), disease severity (β = −0.274 < 0), and demographic characteristics (β = 0.126 > 0) had a significant effect on hospitalization expenses.

**Table 7:** Results of multiple linear regression on hospitalization expenses.

Variables	β	S.E	*t*	*p*-value
Income and security (F1)	0.183	0.007	11.262	0.000
Convenience of medical treatment (F2)	−0.102	0.007	−6.274	0.000
Disease severity (F3)	−0.274	0.007	−16.888	0.000
Demographic characteristics (F4)	0.126	0.007	7.776	0.000

Dependent variable = lg (hospitalization expenses).

#### 3.3.4 Analysis of influencing factors of actual reimbursement rate

To explore the factors influencing the actual reimbursement rate, multiple linear regression was conducted with Factor 1 to 4 used as independent variables and the actual reimbursement rate as the dependent variable. As is shown in Table 8, income and coverage (β = 0.425 > 0), convenience of medical treatment (β = −0.177 < 0), disease severity (β = 0.154 > 0), and demographic characteristics (β = 0.130 > 0) had a significant effect on the actual reimbursement rate.

**Table 8:** Results of multiple linear regression on actual reimbursement rate.

Variables	β	S.E.	*t*	*p*-value
Income and security (F1)	0.425	0.347	28.207	0.000
Convenience of medical treatment (F2)	−0.177	0.347	−11.772	0.000
Disease severity (F3)	0.154	0.347	10.213	0.000
Demographic characteristics (F4)	0.130	0.347	8.614	0.000

Dependent variable = Actual reimbursement rate.

## 4 Discussion

This study explored the current situation and influencing factors of the use of cross-regional hospitalization services in China, taking cross-provincial inpatients from a central province of China as the sample population. The overall sample presented high proportions in sub-groups such as URRBMI, direct settlement, distance to medical institution of 493 km and above, non-tumor diseases, etc. The study found that cross-provincial inpatients more likely chose tertiary hospitals for medical treatment, and their out-of-pocket burden for cross-provincial medical care was heavy. Further analysis indicated that multiple factors jointly affect the utilization of cross-provincial hospitalization services for insured patients.

### 4.1 Higher level of medical institutions was more frequently chosen

The results of this study showed that both the percentage of cross-provincial inpatients choosing tertiary hospitals and the case-average hospitalization expense were higher than the national average level of insured patients in China (89.9% vs. 63.5%, 24,662 yuan vs.10,619 yuan) (20). However, the actual reimbursement rate was lower than the national average level (51.0% vs. 68.1%) (21). Consequently, the out-of-pocket burden on cross-provincial inpatients was heavier. This might be, on the one hand, due to the fact that most of the cross-provincial inpatients were diagnosed with incurable diseases with noticeably high medical costs. They chose tertiary hospitals to seek for better medical resources (7). On the other hand, it might be attributable to the cross-provincial medical referral policy adopted generally in China, which exerted strict control on cross-provincial inpatients, especially on those who did not meet the referral regulations.

### 4.2 Multiple factors combine to influence the utilization of cross-provincial hospitalization services

This study found that multiple factors combined to influence the utilization of cross-provincial hospitalization services among insured patients. Patients with higher incomes tended to choose higher levels of medical institutions, which resulted in great hospitalization expenses Obviously, economic income, to a large degree, determines residents’ affordability for medical services, and seeking for medical treatment is largely a resource-driven behavior (22).

Medical insurance schemes are viewed as a key factor in guaranteeing people’s access to good health care (23, 24). Cross-provincial inpatients insured by UEBMI were more inclined to choose tertiary hospitals than URRBMI patients (92.9% > 88.5%). Although their hospitalization expenses were much higher than that of URRBMI patients, their actual reimbursement rate was also higher. This is mainly because of the large difference in the treatment and reimbursement policy of the two kinds of basic medical insurance schemes. Statistics released by the Bureau of Statistics show that *per capita* contributions of UEBMI were 4,566 yuan in 2020 and *per capita* payments for medical care were 3,734 yuan, while *per capita* contributions of URRBMI were 897 yuan and *per capita* payments were 803 yuan (10). This is similar to the reports by scholars such as Cai (19), Zhang (25), and Zheng (26).

Meanwhile, relevant studies have also shown that medical insurance reimbursement rate and income gap may affect the fairness of health service utilization (27). Thus it is necessary for policy-makers to pay more attention to the needs of patients with low-incomes, especially those insured by URRBMI and traveling across regions for medical care. Potential measures could be to further increase the inpatient reimbursement rate for URRBMI patients, expand the coverage of health service programs, and provide extra medical assistance when necessary. Certainly, narrowing the gap in residents’ income is the most fundamental measure to reduce health inequity. The Government should consider starting with the causes of income gaps among residents and take effective measures to reduce the gap.

Patients, who have more convenient access to medical care, that means short distance to medical institutions and direct settlement of hospitalization expenses, were more inclined to choose higher level medical institutions. As a result, they incurred higher expenses and actual reimbursement rates. On the one hand, this might be due to the fact that patients’ utilization of cross-provincial hospitalization services was influenced by the geographical location of hospitals (4, 28, 29). While pursuing high-quality medical care, patients who have plans to seek cross-provincial medical care mostly tended to choose short-distance cross-regional medical care, considering the non-financial factors such as treatment urgency, transportation and accommodation (30, 31). Fu (32) conducted centrality analysis and spillover effect analysis through social network analysis methods and found that patients who seek medical care across provinces are more likely to move to provinces that are closer and have better medical resources. On the other hand, direct settlement can effectively reduce out-of-pocket economic burden of cross-provincial patients (16) and increase the actual reimbursement rate, but it may exacerbate the “siphon effect” of tertiary hospitals, leading to a significant increase in hospitalization expenses (33, 34). Therefore, it is necessary to improve the coordination level of basic medical insurance, develop a comprehensive system of hierarchical diagnosis and treatment, and explore various medical insurance payment methods to control the rapid rise of medical expenses.

Patients with tumor diseases and more days of hospitalization, i.e., ones with severe diseases, tended to choose higher level medical institutions across provinces. This might be due to the fact that the level of medical treatment in a province could not meet the needs of patients with severe diseases (35). Patients with severe diseases have higher hospitalization expenses and lower actual reimbursement rate, probably because the cross-provincial direct settlement of medical expenses follows the principle called “the medical treatment catalog of the areas providing medical treatment but with the treatment level of the areas where patients are insured.” and the drugs and treatments required by patients with severe diseases may not be included in the care catalogs of the places where medical treatment occurs. Therefore, it is highly necessary to improve the medical services in insured areas, increase the ability of diagnosis and treatment of severe diseases, and narrow the gap in medical technology and treatment level between provinces, so that patients can obtain high-quality medical treatment in their own provinces.

### 4.3 Limitation

Several limitations of this study should be pointed out. Firstly, the data from the basic medical insurance information system were collected in a formative and standardized way, resulting in a limited number of variables related to patient characteristics and utilization of hospitalization services in this study. Despite this, the obtained statistics have covered all the necessary demographic characteristics and hospitalization information to meet the requirements of this study. Secondly, the findings of this study have limited extrapolation to regions with higher level of healthcare resources compared to the sample province, but have applicability to provinces with similar levels of healthcare resources. Finally, this study is a cross-sectional study, and the findings conclude that the identified influencing factors are closely associated with the utilization of hospitalization services, but a causal relationship could not be inferred. Based on the study design, future studies are needed to further explore the causal relationship.

## 5 Conclusion

This study found that there is a higher proportion of cross-regional hospitalized patients to choose tertiary hospitals, incurring heavier out-of-pocket medical burden. Moreover, the utilization of cross-regional hospitalization services could be affected by income, type of insurance, distance to medical care institutions, settlement method, type of disease, and number of days of hospitalization. The findings of this study can be applied to provinces with similar level of medical resources and facing the mutual challenges of the increasing number of cross-regional patients. In the future, it is urgent to strengthen the construction of medical service systems in regions with a large proportion of patients seeking medical care across regions. The role of basic medical insurance scheme should be given full play in guaranteeing people’s access to the medical care they need, so that high-quality medical resources can be fully utilized across regions.

## Data availability statement

The raw data supporting the conclusions of this article will be made available by the authors, without undue reservation.

## Ethics statement

This study was approved by the Biomedical Ethics Committee of Anhui Medical University (No: 83230502). All the surveys and study were conducted in accordance to the relevant guidelines and regulations. All respondents were voluntary and written informed consent was obtained. All data collection is anonymous. We confirmed that all methods of this study were performed in accordance with the Declaration of Helsinki.

## Author contributions

TS was involved in the framework design, review of the article, analysis of the data, writing, and revision of the manuscript. RM and XZ contributed in the literature search and review, and the revision of the article. BL, ZL, and MG critically revised the manuscript. MY participated in extraction of the data. ZT led the conduct of the study and guided the writing process of the article. All authors read and approved the final manuscript. All listed authors have contributed to the development, writing, and revision of this article.
